# Response of coral calcification and calcifying fluid composition to thermally induced bleaching stress

**DOI:** 10.1038/s41598-017-02306-x

**Published:** 2017-05-19

**Authors:** J. P. D’Olivo, M. T. McCulloch

**Affiliations:** ARC Centre of Excellence for Coral Reefs Studies, Oceans Institute and School of Earth Sciences, The University of Western Australia, Crawley, 6009 Australia

## Abstract

Severe, global-scale thermal stress events like those of 1998 and 2016, are becoming more frequent and intense, potentially compromising the future of coral reefs. Here we report the effects of the 1998 bleaching event on coral calcification as well as the composition of the calcifying fluid (cf) from which corals precipitate their calcium carbonate skeletons. This was investigated by using the Sr/Ca, Li/Mg (temperature), and boron isotopes (δ^11^B) and B/Ca (carbonate chemistry) proxies in a *Porites* sp. coral. Following the summer of 1998 the coral exhibited a prolonged period (~18 months) of reduced calcification (~60%) and a breakdown in the seasonality of the geochemical proxies. However, the maintenance of elevated dissolved inorganic carbon (DIC_cf_; >×2 seawater) and pH_cf_ (>8.3 compared to seawater ~8.0) even during severe stress of 1998 indicate that a minimum threshold of high aragonite saturation state (Ω_cf_) of ~14 (~×4 seawater), is an essential pre-requisite for coral calcification. However, despite maintaining elevated levels of Ω_cf_ even under severe stress, coral growth is still impaired. We attribute this to reductions in either the effective active volume of calcification and/or DIC_cf_ as bleaching compromises the photosynthetically fixed carbon pool available to the coral.

## Introduction

Globally, corals are being subject to more frequent and severe bleaching events^1, 2^ as the oceans warm due to CO_2_ induced climate change. Coral bleaching has been directly linked to enhanced thermal stress, manifested as loss of symbionts, reduced rates of growth (calcification), and associated partial to whole colony mortality^3–6^. The response of calcifying organisms, such as corals to regimes of increased ocean temperature and lower seawater pH (e.g. IPCC^7^) will largely depend on their ability to sustain sufficiently high rates of calcification to counter the effects of increased coral reef degradation and bio-erosion. Coral calcification is a physiological mediated process that occurs in a semi-isolated environment of the calcifying fluid (cf) in the sub-calicoblastic space^8, 9^. The maintenance of elevated pH and dissolved inorganic carbon concentration at the calcification site and hence aragonite saturation state (pH_cf_, DIC_cf_ and Ω_cf_ respectively) are key mechanisms used by corals to promote the precipitation of their calcium carbonate skeleton^10–12^. It is therefore critical to study how sub-optimal conditions of coral calcification associated with thermal stress events are manifested in the physiological controls that corals exert on the carbonate chemistry of their calcifying fluid.

Trace element ratios (TE’s) Mg/Ca, Sr/Ca, and Li/Mg measured in the carbonate skeleton of corals have typically been used to reconstruct seawater temperature^13–15^, as well as providing important information about the chemical conditions required for biomineralization^16, 17^. For example, Sr/Ca is not only a sensitive proxy for seawater temperature, but also dependent on changes in the activity of the Ca^2+^ATPase and associated kinetic effects (Rayleigh fractionation)^17^. For instance, anomalous Sr/Ca coral values have been reported during periods of thermal stress^18–20^ which Marshall and McCulloch^20^ attributed to the inhibition of Ca^2+^ATPase. The potential of TE’s as bleaching indicators; however, has not yet been fully explored, although after a short-term (~6 weeks) bleaching experiment no consistent response was found for coral Sr/Ca, Mg/Ca, U/Ca and Ba/Ca ratios amongst different species^21^. Early work based on skeletal coral δ^18^O or δ^13^C suggested that these proxies could be used to identify and characterize bleaching events^22–25^. However, kinetic isotope effects associated with growth rates mean that these proxies cannot be unequivocally used to identify past bleaching events^21, 26^.

Boron isotopes (δ^11^B), initially considered a seawater pH proxy^27^, reflects the pH of the calcifying fluid, which in corals can be influenced by both the external environment as well as physiological controls (e.g. H^+^ pumping, DIC transport)^10, 28^. Here we examine the combined δ^11^B-B/Ca systematics in corals as a potential indicator of bleaching events due to the likely control of aragonite saturation state (Ω_cf_) on calcification and its dependence on pH_cf_ and particularly bleaching induced variation in DIC_cf_^29, 30^. Bleaching induced reductions in the rate of calcification^5, 6^ may then be expected to result in distinctive changes in pH_cf_ and/or DIC_cf_. Experimental studies on bleached corals show disparate outcomes ranging from no significant differences in the δ^11^B signature between bleached and control corals^21^ to a negative δ^11^B anomaly and hence lower pH_cf_ associated with thermally stressed corals^31^. However, as mentioned the response to thermal stress would likely depend on the combined factors controlling the pH_cf_ as well as the DIC_cf_ (e.g. photosynthesis/respiration, allocation of carbon and active transport) and how these respond to the specific level of stress. Thus to adequately understand the effects of thermal stress events it is clear that a more complete understanding of the full carbonate chemistry at the site of calcification is required, that is both the pH as well as the DIC.

To date attempts to constrain changes in the carbonate chemistry of the calcifying fluid have mainly focused on combining the information from TE’s and δ^11^B^17, 32, 33^. In this regard new inorganic experiments^34^ have demonstrated the feasibility of determining the carbonate ion concentration and hence the DIC of the calcifying fluid from the combined B/Ca and δ^11^B systematics. Together with constraints of the pH from δ^11^B ratios this now allows the complete carbonate chemistry of the calcifying fluid to be determined more reliably^12^. Here we investigate the changes in the carbonate chemistry of the calcifying fluid of massive *Porites* coral from the Great Barrier Reef (GBR) subject to acute but non-lethal thermal stress during the global bleaching event of 1998. We utilise growth data, and skeletal δ^11^B and trace element data compositions, including B/Ca, to quantify the concomitant changes in both pH_cf_ as well as DIC_cf_ during bleaching. The geochemical information is thus used to reconstruct the chemical changes at the site of calcification that occur during and following thermal stress events. Furthermore, we also evaluate the potential of these biogeochemical tracers as indicators of past bleaching events.

## Methods

### Sample collection and age model

**Figure 1:**
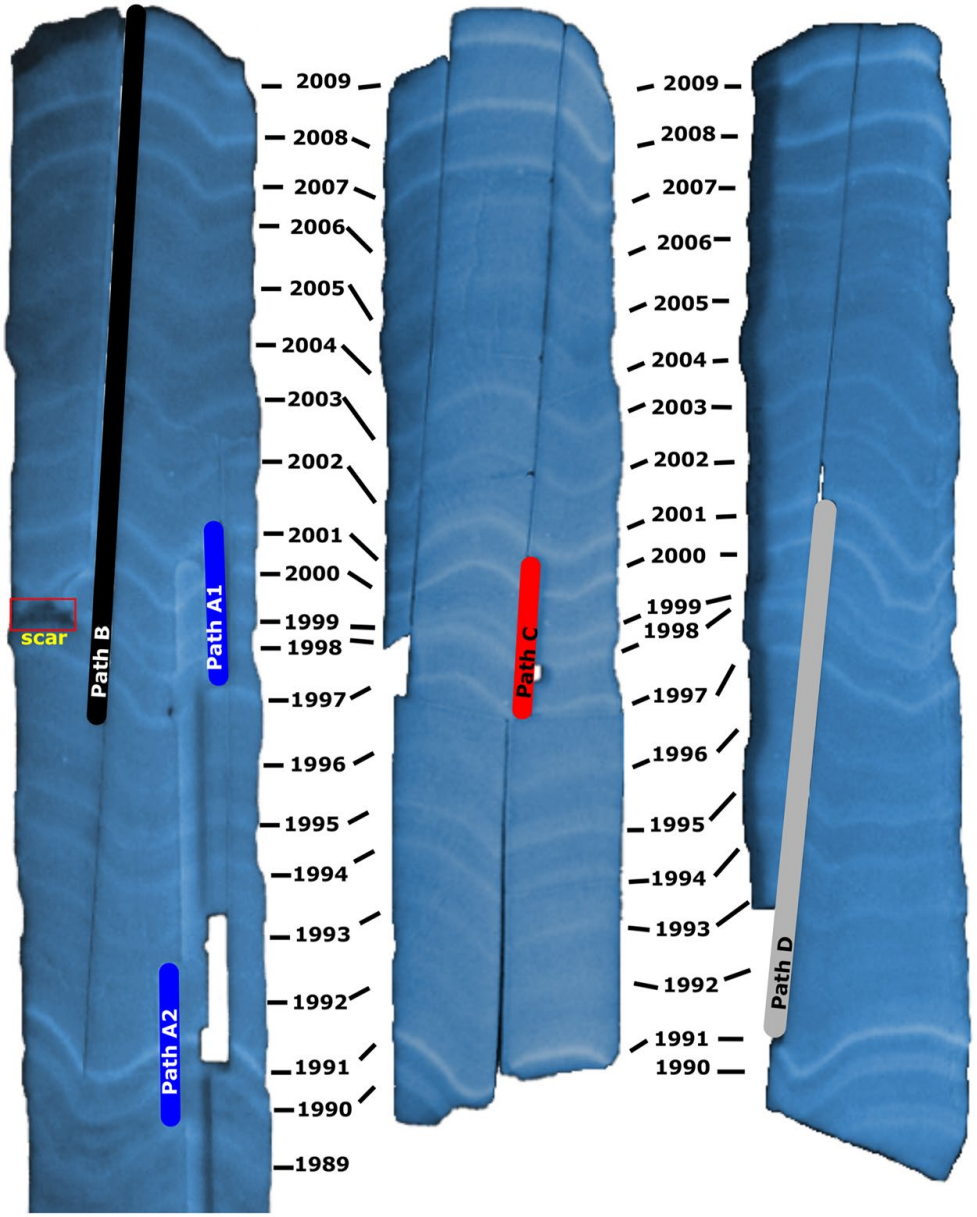
Slabs from core HAV09_3 displaying annual luminescent lines revealed under UV light. Sample paths for geochemical analysis are indicated; the presence of a small scar associated to the summer of 1998 is also highlighted. The coloured area under the scar correspond to coral tissue that died around 1998, the associated scar it self is only a few microns thick.

In July 2009 a coral core was collected from a living *Porites* colony adjacent to Havannah Island (18° 50.271′ S, 146° 31.922′ E) in the inshore region of the central GBR. The top of the colony was in 3.6 meters of water, 5.8 meters at the bottom. The coral core was sliced along the maximum growth axis in 7 mm thick slices. Luminescent lines revealed under UV light (Fig. 1) and density bands from X ray images (Supplementary Fig. S1) were used to establish a general chronology and sample intervals. The distinctive patterns observed in the luminescent bands associated with flood events^35^ served as an excellent control of the chronology. These luminescent lines were also used to calculate linear extension^5^. The chronology was fine-tuned with the information from the Sr/Ca trace element ratio using *in situ* temperature data as a reference.

### Geochemical analyses

Coral slabs were bleached in 6% reagent grade NaClO for 24 hours to remove organic material and rinsed three times in an ultrasonic bath filled with DI water. Powder samples were milled along four paths for geochemical analysis (Fig. 1). The main difference between sampled paths is that path B was sampled closer to the scar. Path A was sampled at bimonthly resolution (~6 samples per year) for δ^11^B from 1989–1992 and 1997–2000. Path B, sampled at bimonthly resolution (~7 samples per year), was analysed for TE’s (1997–2009) and δ^11^B (1997–2002). Path C, sampled at better than monthly resolution (~14 samples per year) from 1997–2002, was analysed for TE’s and δ^11^B. Path D, sampled at better than bimonthly resolution (~10 samples per year) from 1991–2000, was analysed for TE’s and δ^11^B. Powder samples from path A were treated with hydrogen peroxide (30%) to remove organic material prior to sample separation. About 20 mg (±0.5 mg) of coral sample was used for samples from path A and, following methodological improvements, 10 mg (±0.25 mg) for samples from paths B, C and D. All samples and standard solutions were prepared using distilled HNO_3_ and MQ water (18.2 MΩ).

#### Trace elements

Samples from paths B, C and D were analysed for trace elements at the University of Western Australia (UWA) following methods described by Holcomb, *et al*.^36^. Sample preparation was undertaken in a metal-free HEPA-filtered clean room. Sub-samples of ~10 mg of coral powder were weighed and dissolved in 0.46 mL of 0.58 N HNO_3_. A 30 µl aliquot was diluted in 2970 µl 2% HNO_3_ (100 ppm Ca) for trace element analysis by Quadrupole-ICP-MS while 400 µl from the remaining original solution was used for boron isotopes analysis by MC-ICP-MS (see following section).

The 100 ppm Ca solution was used for ^7^Li, ^25^Mg and ^11^B analysis. A sub-aliquot of 300 µl was taken from the 100 ppm Ca solution and diluted in 2700 µl 2% HNO_3_ (10 ppm Ca) containing a spike solution with ~19 ppb ^45^Sc, 19 ppb ^89^Y, 0.19 ppb ^141^Pr, and 0.095 ppb ^209^Bi. The 10 ppm Ca solution was used for the determination of ^25^Mg, ^43^Ca, ^86^Sr and ^238^U. All trace element measurements were undertaken using a Q–ICP-MS (X-series II, Thermo Fisher Scientific; UWA). Long-term reproducibility of TE/Ca derived from repeated analyses of the NEP coral powder (an in-house standard) that underwent full chemical preparation resulted in Mg/Ca = ±2.2%, Sr/Ca = ±0.4%, and U/Ca = ±1.2% (2s RSD; n = 67). Long-term reproducibility for the NEP in-house standard (2s RSD; n = 25) was ±1.4% for Li/Mg and ±5% for B/Mg data. The B/Mg data was combined with the Mg/Ca data to estimate B/Ca to minimise uncertainties associated with the large dynamic range of Ca concentrations.

#### Boron isotopes

Samples from paths B, C and D were analyzed by MC-ICP-MS using a NU Plasma II (UWA) while samples from path A were analyzed by positive thermal ionization mass spectrometry (PTIMS) using a Thermo Finnigan TRITON (Australian National University). For all samples boron was purified using ion chromatography prior analysis. The boron separation technique used for PTIMS is based on methodology by Wei, *et al*.^37^ refined by Trotter, *et al*.^38^. For MC-ICP-MS, a combined cation/anion ion-exchange technique was employed^39^. Long-term averages (>1 year) for NEP B analyses are 26.35 ± 0.44‰ (2 SD, n = 33) using PTIMS, and 25.96 ± 0.32‰ (2 SD, n = 70) using MC-ICP-MS. This offset is consistent with the +0.45‰ offset for coral samples (n = 8) analysed by PTIMS relative to the MC-ICP-MS data^40^. To maintain consistency between the two data sets a −0.45‰ correction was applied to the PTIMS data. We opted to correct the PTIMS data as the MC-ICP-MS measurements of the international carbonate standards JCp-1 gave a δ^11^B value of 24.36 ± 0.34‰ (2 SD; n = 101), which agree with the previously reported values of 24.33 ± 0.11‰ (SE)^41^.

Conversion of coral δ^11^B to pH of the calcifying fluid (pH_cf_) values was undertaken using the standard relationship:

1 $${{\rm{pH}}}_{{\rm{cf}}}={\rm{p}}{K}_{{\rm{B}}}^{\ast }-\,{\rm{log}}[\frac{{\delta }^{{\rm{11}}}{{\rm{B}}}_{{\rm{SW}}}-{\delta }^{{\rm{11}}}{{\rm{B}}}_{{\rm{carb}}}}{{\alpha }_{{\rm{B3}}\text{-}{\rm{B4}}}{\delta }^{{\rm{11}}}{{\rm{B}}}_{{\rm{carb}}}-{\delta }^{{\rm{11}}}{{\rm{B}}}_{{\rm{SW}}}+{\rm{1000}}({\alpha }_{{\rm{B3}}\text{-}{\rm{B4}}}-{\rm{1}})}]$$

where δ^11^B_SW_ is the B isotope composition of seawater (δ^11^B_SW_ = 39.61‰)^42^, α_B3-B4_ is the B isotope fractionation factor (α_B3-B4_ = 1.0272)^43^ and δ^11^B_carb_ is the B isotope composition of the coral. The logarithm of B dissociation constant (p*K* ^*^_B_)^44^ was adjusted to the ambient temperature and salinity^38^ based on instrumental data described below^40^. All pH values are expressed on the total scale (pH_T_).

### Composition and fractionation of the calcification fluid

To quantify the changes in the aragonite saturation state (Ω_cf_) and dissolved inorganic carbon (DIC_cf_) of the calcifying fluid we first modelled the changes in TE’s in the coral based on closed system Rayleigh fractionation equations following the definitions used by Sinclair^45^:

2 $${(\frac{{\rm{T}}{\rm{E}}}{{\rm{C}}{\rm{a}}})}_{{\rm{a}}{\rm{r}}{\rm{a}}{\rm{g}}}={(\frac{{\rm{T}}{\rm{E}}}{{\rm{C}}{\rm{a}}})}_{{\rm{c}}{\rm{f}}}\frac{(1-{P}^{{{\rm{K}}{\rm{d}}}_{{\rm{T}}{\rm{E}}}})}{(1-P)}$$

where (TE/Ca)_cf_ is the TE/Ca ratio of the calcifying fluid, (TE/Ca)_arag_ is the TE/Ca ratio of the skeleton, *P* is the proportion of Ca remaining in the calcifying fluid after precipitation has ended (precipitation efficiency) and Kd_TE_ is the distribution coefficient of the TE/Ca between the crystal and the solution. Similar equations were utilised for Sr/Ca and Mg/Ca; for Sr/Ca Kd_Sr_ = *e* ^(−1.86+600/T^_K_^)^^45^ and for Mg/Ca Kd_Mg_ = *e* ^(−13+1700/T^_K_^)^, where T_K_ is the temperature in Kelvin. The coefficient for the Kd_Mg_ was adjusted to match the coral data^15^ while ensuring *P* was constrained between 0 and 1, and [Ca] in the cf was higher than seawater (e.g. >10.25 mmol). The selected Kd_Mg_ coefficient is within the reported uncertainty^45^ of the temperature dependence of Kd_Mg_ from partitioning experiments^46^. This produced two independent equations 2 using the coral Sr/Ca and Mg/Ca elemental ratios with two unknowns (Ca_cf_ and *P*). These system of equations were solved for Ca_cf_ and *P* to match each pair of coral Sr/Ca and Mg/Ca data (see supplementary). For the model it was assumed that Ca^2+^ was transported to the site of calcification by two mechanisms: (1) derived directly from seawater and (2) pumped to the site of calcification by an enzyme system (Ca^2+^ATPase)^8^; and therefore enriched in the cf compared to seawater. Although still a matter of debate^17, 47, 48^, it was assumed that the pump was specific for Ca^2+^ only. The lower Sr/Ca measured in coral compared to inorganic precipitation is consistent with the view that the pump is specific for Ca^2+^ rather than both Sr^2+^ and Ca^2+^ ^17^. For simplicity and considering the integrated < monthly resolution nature of our sampling it is assumed that conditions are modified before calcification occurs and then operates as a closed system.

#### Carbonate-ion concentration

The coral B/Ca was used to estimate [CO_3_^2−^] in the cf based on the assumption that B/Ca ratio in the aragonite is a function of the substitution of [B(OH)_4_^−^] with [CO_3_^2−^], as shown by inorganic experiments^34^. In this study we used the equation described by McCulloch, *et al*.^12^ which is a simplification of the equations described by Holcomb, *et al*.^34^ with the concentration of B in the cf being mainly controlled by [CO_3_^2−^] substitution and therefore similar to that in natural seawater. We thus have:

3 $$[{{\rm{CO}}}_{{\rm{3}}}^{2-}]=(2.965\times {10}^{-3}\ast {\rm{e}}{\rm{x}}{\rm{p}}(-\,0.0202\ast [{{\rm{H}}}^{+}]))\ast (\frac{[{\rm{B}}{({\rm{OH}})}_{{\rm{4}}}^{-}]}{{\rm{B}}/{\rm{Ca}}})$$

where [H^+^] is the concentration of H^+^ in the cf in nmol/kg estimated from coral δ^11^B derived pH_cf_ and [B(OH)_4_^−^] is calculated from the relationship:

4 $$[{\rm{B}}{({\rm{OH}})}_{{\rm{4}}}^{-}]=\frac{{{\rm{B}}}_{{\rm{T}}}}{(1+\frac{[{{\rm{H}}}^{+}]}{{K}_{{\rm{B}}}^{\ast }})}\ast {\rm{1000}}$$

where B_T_ is the boron in seawater in mmol/kg estimated from salinity (B_T_ = (0.4326 mmol/kg)*(S/35))^49^; and *K*_B_^*^ is the stoichiometric equilibrium constant of boric acid^44^, as described in equation 1.

With the estimates of pH_cf_ and [CO_3_^2−^] it is then possible to estimate the remaining carbonate parameters in the calcifying fluid. From the δ^11^B-reconstructed pH_cf_ and B/Ca-reconstructed [CO_3_^2−^] we estimated [DIC_cf_] based on the relationship:

5 $${\rm{DIC}}={{\rm{CO}}}_{3}^{2-}(1+\frac{[{{\rm{H}}}^{+}]}{{K}_{2}^{\ast }}+\frac{{[{{\rm{H}}}^{+}]}^{2}}{{K}_{1}^{\ast }{K}_{2}^{\ast }})$$

where *K* ^*^_1_ and *K* ^*^_2_ are the first and second acidity constants of carbonic acid, respectively. Calculations were made using CO2SYS with carbonate constants *K* ^*^_1_ and *K* ^*^_2_ from Mehrback *et al*.^50^ refitted by Dickson and Millero^51^, and for sulfate from Dickson^44^ with 0 dbar pressure.

Finally, the modelled [Ca^2+^] (from TE’s) and [CO_3_^2−^] (from δ^11^B and B/Ca) were used to estimate Ω_cf_ according to the relationship:

6 $${\rm{\Omega }}=\frac{[{{\rm{C}}{\rm{a}}}^{2+}]\ast [{{\rm{C}}{\rm{O}}}_{3}^{2-}]}{{K}_{{\rm{s}}{\rm{p}}}^{\ast }}$$

where *K* ^*^_sp_ is the solubility constant for aragonite as a function of temperature and salinity^52^.

### Instrumental records and temperature calibrations

An inner-shelf *in situ* seawater temperature record was reconstructed using logger data from Havannah Island (2009–2013) and extended back to 1992 with logger data from the nearby sites of Pandora Reef, Magnetic Island and the Greater Palm group (AIMS, http://data.aims.gov.au/, 2016). The *in situ* data was extended back to 1989 using satellite sea surface temperature (SST) centred at 18.5 °S and 146.5 °E with a resolution of 1° × 1° from NOAA OI SST v2 (iridl.ldeo.columbia.edu; 2014). The OI SST v2 data was scaled to match the amplitude and average of the inner-shelf *in situ* data. Both geochemical and temperature records, were re-sampled to bimonthly resolution using linear interpolation with the Analyseries 2.0.8 software^53^. Pearson product-moment correlation coefficients were calculated to evaluate the relationships between the proxies and temperature. River discharge data was obtained from the Department of Natural Resources and Mines (DNRM) Water Monitoring Information Portal (WMIP) for the Burdekin River (https://water-monitoring.information.qld.gov.au/; 2016). Seasonal variation in salinity was estimated based on the linear relationship between the magnitude of past flood events and corresponding salinity values^40^. Photosynthetically active radiation from the central GBR was obtained from several locations in the central GBR (Davies Reef, Orpheus Island, Magnetic Island Hardy Reef John Brewer Reef, Myrmidon Reef, Rib Reef and Cape Bowling Green; AIMS, http://data.aims.gov.au/, 2016).

## Results

### Linear extension rates

**Figure 2:**
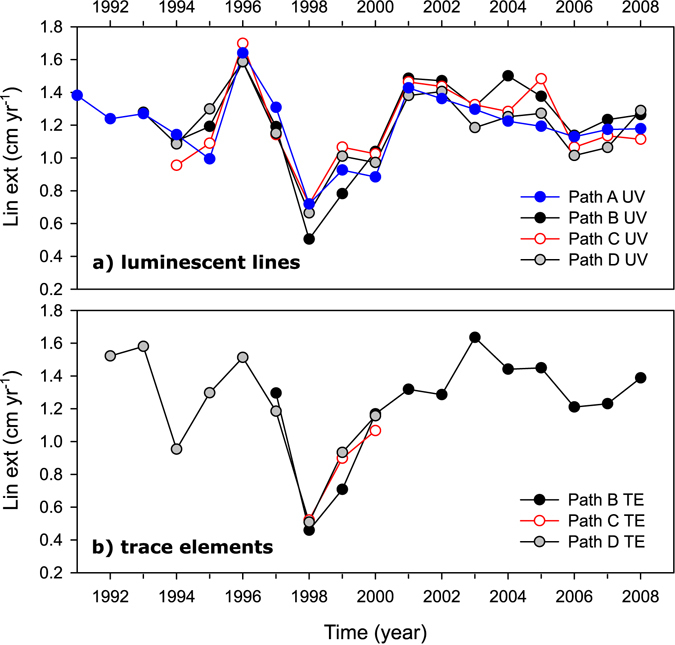
Annual records of skeletal growth measured as linear extension from: (**a**) luminescent lines sampled along all four sample paths and (**b**) TE ratios sampled along three different paths. Path B and Path D were sampled along the axis maximum growth axis but from different slabs, with Path B sampled closer to a small scar observed during the summer of 1998 (Fig. 1). Path A and Path C were sampled along the same sub-optimal growth axis but different slabs.

Annual coral growth records for core HAV09_3 were characterized by a reduction in linear extension of >60% in 1998, with a full recovery by 2000 (Fig. 2). The decrease in extension rate was consistent for the geochemical and luminescent bands data along all sampled paths. The main difference between sample paths was a longer recovery time for path B. The small scar observed on the edge of the core adjacent to path B (Fig. 1), where the growth bands corresponding to 1998 and 1999 merged, suggests that the coral also suffered partial mortality.

### Trace elements

**Figure 3:**
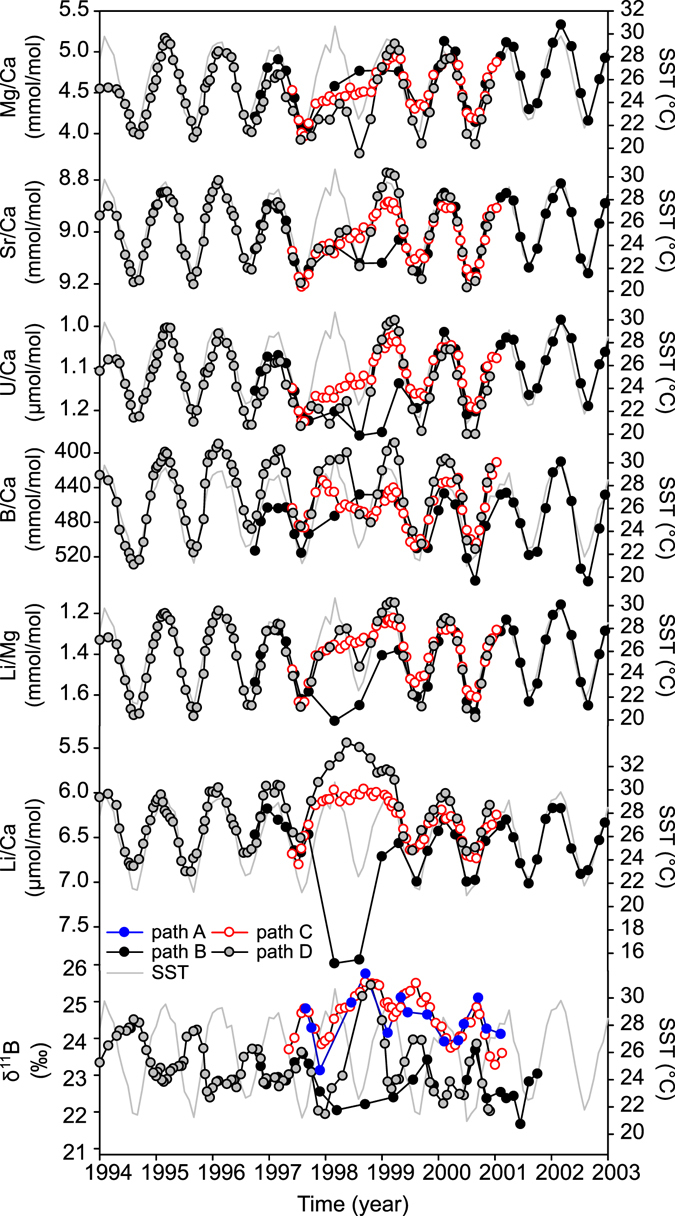
Untreated (uneven time-distance) trace element and boron isotopes records centred around 1998 for the four different sample paths (see Fig. 2) compared to monthly SST. The records in their full length are presented in the supplementary material.

**Figure 4:**
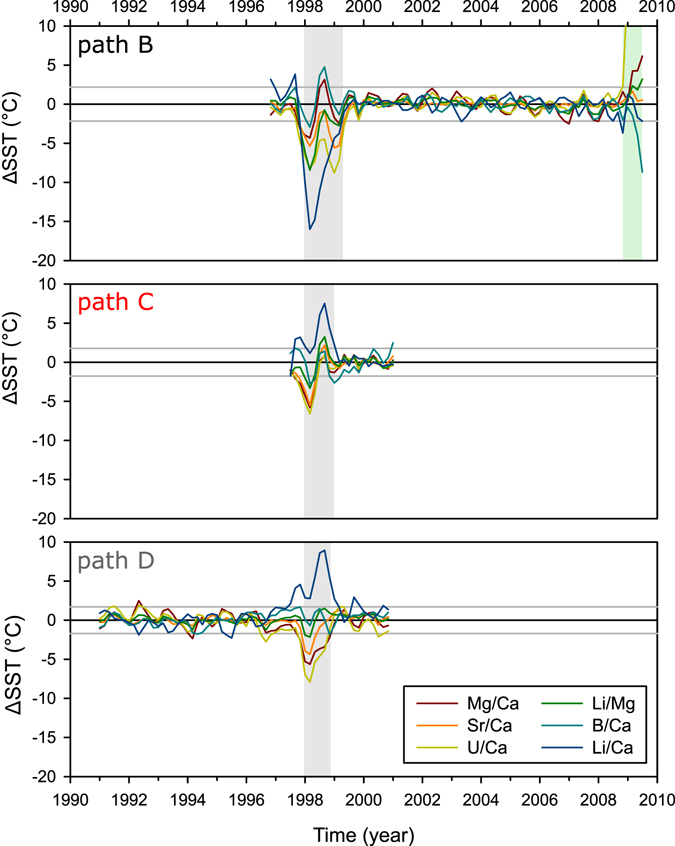
Difference between the bimonthly (interpolated) records of measured SST and TE-seawater temperature reconstruction for three different sample paths (see Fig. 2). The period covered by the tissue layer is highlighted in green while the period affected by the 1998 thermal stress event is highlighted in grey. The horizontal grey lines indicate the average 2 SD for all elements, this is approximate a 2 °C difference.

The TE signals in core HAV09_3 measured along path B showed clear seasonal patterns (Fig. 3 and Supplementary Fig. S2) generally in good agreement with seawater temperature changes, with the highest correlation observed for Sr/Ca and Li/Mg (Table 1). A breakdown in the seasonal TE’s signal, with a general initial enrichment for most elements with respect to calcium, coincides with the 1998 thermal stress event and extends into at least the beginning of winter of 1999, approximately 18 months (Fig. 3). The magnitude of the TE/Ca anomaly varied between proxies, translating into temperature anomalies that ranged from 4 °C for Mg/Ca to >15 °C for Li/Ca (Fig. 4). The analysis of parallel sample paths from separate slabs (paths C and D) revealed a similar pattern following the 1998 thermal stress event, characterized by a breakdown of the seasonal TE’s cycle (Fig. 3). However, the TE’s and coral growth data suggested that the recovery occurred faster, within approximately one year, along paths C and D compared to path B. Boron was in general the TE/Ca least affected by the thermal stress event (Fig. 4). For Li/Ca the temperature anomaly changed from being negative along sample path B to positive along sample paths C and D.

**Table 1:** Pearson correlation coefficients (r) and corresponding p-values for the relationships between TE’s and δ^11^B coral bimonthly records with the bimonthly seawater temperature record.

		Mg/Ca	Sr/Ca	U/Ca	Li/Mg	B/Ca	Li/Ca	δ^11^B
Path B	r	0.913	−0.936	−0.885	−0.964	−0.887	−0.838	−0.503
(n = 65)	p	<0.001	<0.001	<0.001	<0.001	<0.001	<0.001	0.015
Path C	r	0.935	−0.937	−0.912	−0.934	−0.865	−0.817	−0.476
(n = 16)	p	<0.001	<0.001	<0.001	<0.001	<0.001	<0.001	0.063
Path D	r	0.905	−0.973	−0.899	−0.963	−0.921	−0.839	−0.596
(n = 52)	p	<0.001	<0.001	<0.001	<0.001	<0.001	<0.001	<0.001
Average	r	0.918	−0.949	−0.899	−0.954	−0.891	−0.831	−0.525

The anomalous data associated with the 1998 thermal stress event was not included in the calculations.

### Calcium in the calcifying fluid

**Figure 5:**
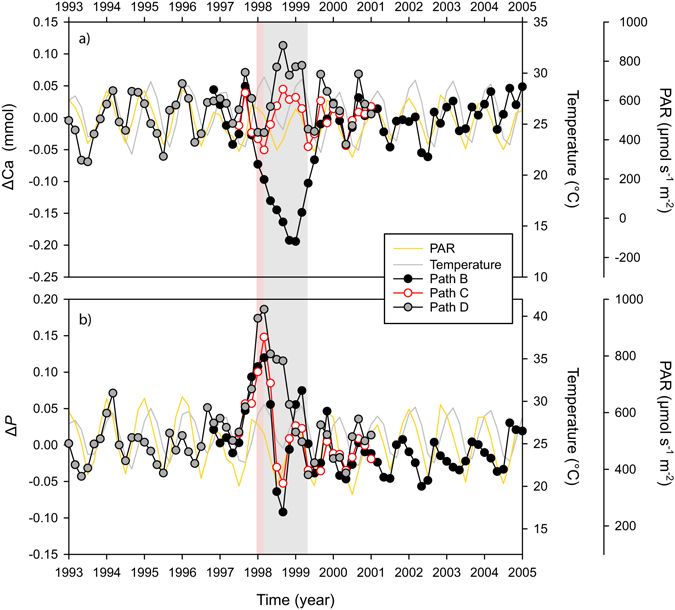
Bimonthly (interpolated) anomalies (Δ) for the modelled changes in: (**a**) the concentration of Ca^2+^ in the calcifying fluid ([Ca^2+^]_cf_) and (**b**) the proportion of Ca^2+^ remaining in a batch of calcifying fluid after calcification finalizes (*P*). Anomalies (ΔCa or Δ*P*) were estimated by subtracting the expected seasonal variation due to temperature from the modelled data. The temperature expected values were based on the inverse relationships of *P* and [Ca^2+^]_cf_ with temperature (Supplementary Fig. S3). First the expected (predicted) *P* and [Ca^2+^]_cf_ from temperature changes alone were estimated using the corresponding equations in the supplementary Table S1. Then the resulting predicted values were subtracted from the *P* or [Ca^2+^]_cf_ values modelled from the trace elements Sr/Ca and Mg/Ca. The pink shaded area highlights the timing of the 1998 bleaching event, the grey shaded area highlights the recovery period. Seawater temperature and PAR (light) are shown for comparison purposes.

The [Ca^2+^]_cf_ showed seasonality in antiphase with temperature (Supplementary Fig. S3), with no consistent response in [Ca^2+^]_cf_ observed following the summer of 1998 along different sample paths (Fig. 5). Path B showed a negative anomaly during the summer of 1998 returning to original baseline values during the winter of 1999. Path C showed no clear change associated to the 1998 event while path D showed a positive anomaly during the winter of 1998. The modelled proportion of Ca remaining in the cf after calcification was completed (*P*) data (Supplementary Fig. S3) showed seasonality in phase with changes with temperature, but during the summer bleaching of 1998 exhibited a positive anomaly along the three sampled paths (Fig. 5). This positive anomaly was most severe along path D, with values remaining high during all of 1998 and part of 1999 suggesting a decrease in precipitation efficiency (e.g. less calcium precipitated from a batch of calcifying fluid). The positive anomaly in Path B and C quickly shifted into a negative anomaly just after the winter of 1998, returning to “normal” values at the end of 1998. After removing the effect from temperature from the modelled [Ca^2+^]_cf_ and *P* records (Δ*P* and ΔCa) an apparent seasonality was still present in the resulting records (Fig. 5). We tested if Δ*P* and ΔCa were correlated with river discharge and photosynthetically active radiation (PAR) a light indicator, with PAR showing the highest correlations (r = 0.34, 0.38 and 0.25 for Δ*P* and r = 0.33, 0.30 and 0.33 for ΔCa for path B, C and D, respectively). However, the relation with PAR was only significant (p < 0.05) for ΔCa along path D (r = 0.33; n = 54; p = 0.01).

### δ^11^B and B/Ca constraints on the pH and DIC of the calcifying fluid pH

**Figure 6:**
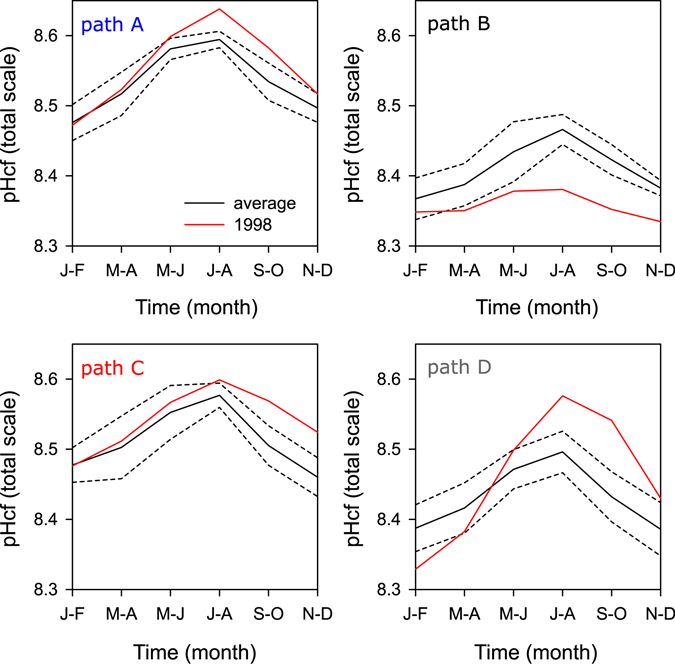
Bimonthly (interpolated) mean climatology in coral δ^11^B-pH_cf_ (black line) compared to the seasonal variability observed during 1998 (red line) for four different sample paths (see Fig. 2), the dashed lines represent one standard deviation from the mean value.

**Figure 7:**
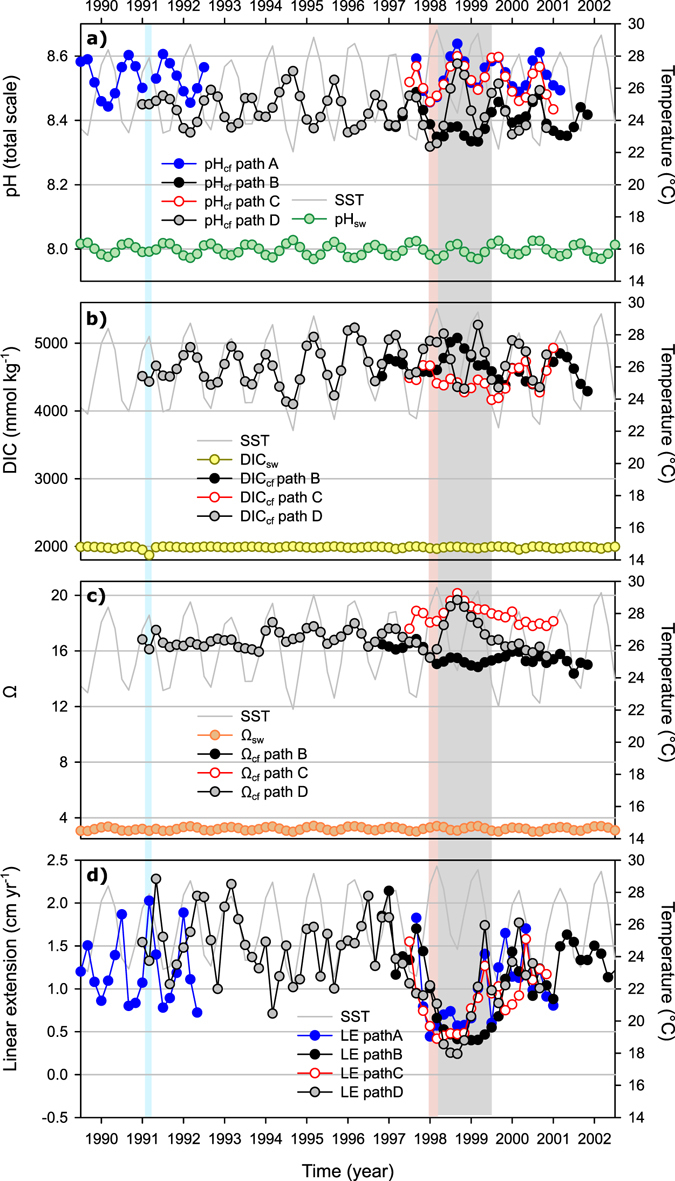
Coral bimonthly (interpolated) reconstructions of (**a**) pH_cf_, (**b**) DIC_cf_, (**c**) Ω_cf_ and (**d**) linear extension (calculated from trace element age model) compared against modelled seawater pH (pH_SW_), seawater inorganic carbon (DIC_SW_) and seawater aragonite saturation state (Ω_SW_), respectively. SST records are shown in all panels for comparison purposes. The bimonthly pH_SW_, DIC_SW_ and Ω_SW_ records were modeled according to D’Olivo, *et al*.^40^ (see supplementary). The pink shaded area highlights the timing of the 1998 bleaching event, the grey shaded area highlights the recovery period and the blue shaded area highlights the timing of the 1991 very large flood event. The increase in pH_cf_ during the 1991 could be explained by the decrease in DIC_cf_ following the dynamic control of the cf described in the text.

All sample paths showed clear δ^11^B seasonal cycles with an average amplitude of typically >1‰ (Fig. 3 and Supplementary Fig. S2), with lower isotopic values and hence lower pH_cf_ associated with summer (Fig. 6). A clear difference was observed however in the δ^11^B baseline values for the different sample paths. One-way ANOVA (Holm-Sidak all pairwise multiple comparisons) over the common period for all paths (excluding 1998–1999) indicate that δ^11^B values for path A and C were significantly different (p < 0.05, n = 21) from values from path B and D. For paths A and C, δ^11^B averaged 24.5‰, while paths B and D had a lower average δ^11^B value of 23.2‰. The δ^11^B values translate to an average pH in the calcifying fluid (pH_cf_) of 8.55 (path A and C) and 8.45 (path B and D) respectively, with typical seasonal amplitude in pH_cf_ of 0.1 to 0.15 units (Figs 6 and 7). Two distinctively different responses were observed during the 1998 thermal stress event. Paths B and D showed a negative anomaly during the summer of 1998 which for path B persisted for the entire year. In contrast, paths A and C showed no clear change during the 1998 stress period (summer). During the recovery period (May-June 1998 to November-December 1998) paths A, C and D were characterized by marked positive excursions in pH_cf_, with values being higher than those observed for the entire record.

On average DIC (~4500 mmol/kg) and Ω (~17) in the cf were elevated relative to seawater values (Fig. 7) by more than two and four times, respectively. As with pH_cf_, differences were observed between the baseline Ω_cf_ values from different sample paths with values being ~2 units higher for path C, compared to path B and D. A clear seasonal variability was observed in the reconstructed DIC_cf_ and, less evidently, Ω_cf_ that were in phase with changes to SST, that is higher values in summer and lower values in winter. For δ^11^B (pH), the opposite behaviour was found with lower values in the summer. The amplitude of the pH_cf_, DIC_cf_ and Ω_cf_ seasonal variability was several times larger in comparison to the seasonal amplitude in seawater (>2, >30 and >5, respectively). Path D showed a minor reduction in DIC_cf_ during the summer 1998, recovering before the onset of the 1998 winter (Fig. 7b). For path B DIC_cf_ showed a reversal of the seasonal variability with lower summer values, and higher winter values after the thermal stress event of 1998 until the end of the summer of 1999. For path C, DIC_cf_ was characterized by small summer negative anomaly during 1998 and apparent suppression of the full seasonal amplitude until returning to normal values at the end of the summer of 1999.

During the summer of 1998 a reversal in seasonality was observed for Ω_cf_, with all paths showing lower values compared to summer values during normal (non-bleaching) years (Fig. 7c). Path B showed the largest negative anomaly, with values staying low (<16) throughout 1998 and most of 1999, but still highly elevated in respect to seawater (~4 × Ω_SW_). Path C and D showed a small anomaly during the summer of 1998 with values remaining similar to winter values, this was followed by a large positive anomaly during the winter of 1998 with values returning to normal at the end of the summer of 1999. The recovery phase after the stress event of 1998 for paths A, C and D were characterized by high pH_cf_ and Ω_cf_ values, despite the contemporary low growth rates. This was particularly evident along path D where the peak in pH_cf_ and Ω_cf_ coincided with the lowest rates of extension. The data from path D also revealed that a limited duration anomaly occurred in 1991 that was characterized by higher summer pH_cf_ and lower Ω_cf_ and DIC_cf_, coinciding with the largest flood event covered by the coral record.

## Discussion

### The 1998 bleaching event

In the central GBR, the summer of 1998 is the warmest yet recorded, with the average monthly seawater temperature in February reaching 31.1 °C on the inshore reefs (Supplementary Fig. S5), 1.6 °C higher than the ‘normal’ maximum monthly average temperature. The inshore *in situ* SST data indicates that 10 Degree Heating Weeks (DHW) were exceeded during the summer of 1998, this is by far the highest temperature anomaly observed in the 24 years since data became available (Supplementary Fig. S6). As a result of this thermal stress event in the inner-shelf central GBR more than 60% corals bleached^54^ and the calcification rates of massive *Porites* corals was significantly reduced^5, 6^. For the *Porites* coral analysed here, clear evidence of stress was observed in the form of scaring, >50% reduction in growth (Fig. 2) and a breakdown in the seasonality of the TE’s during 1998 (Fig. 3), with a full recovery only after two years.

### Trace elements as proxies of thermal stress events

Despite the limitations of U/Ca, Mg/Ca and Li/Ca as seawater temperature proxies (e.g. lower correlation with SST than Sr/Ca or Li/Mg; Table 1), these appear to act as sensitive proxies for past thermal stress events (Fig. 4). The sensitivity of Li, U and Mg to stress events is not surprising as these elements have been reported to be affected by coral physiology, particularly growth and calcification^15, 33, 55^. Li/Ca appears to be particularly useful as a stress indicator as it not only showed the largest anomalies but also a distinctive response depending on the degree of stress. Positive (colder temperatures) anomalies were associated with near death conditions (Fig. 4, path B) while negative (warmer temperature) anomalies were associated with less severe stress (Fig. 4, paths C and D). Although Li/Ca and Mg/Ca ratios appear to be primarily controlled by growth rates^15, 56^, the dissimilarity in the response of these ratios following the 1998 event points to differences in the incorporation mechanisms of these elements under stress. Given the proximity of path B to the scar it cannot be completely discarded that the two samples of path B showing the larger Li/Ca anomalies could have been affected by diagenetic material incorporated during formation of the bleaching scar^57^. However, this is considered unlikely since the remaining samples from along the same path (B), while still corresponding to the recovery phase but removed from the scar area, also show anomalies of the same sign although of a lesser magnitude (e.g. positive Li/Ca anomaly, negative δ^11^B anomaly; Fig. 3).

The difference in the magnitude of the anomaly and recovery time observed in the geochemical data along multiple paths associated with the 1998 event (Fig. 3) suggests different intra-colonial degrees of stress. For example, the magnitude of the anomaly in the TE’s associated with the 1998 stress event was in general more severe for path B, sampled close to a region showing partial mortality (Fig. 4). The recovery interval for path B represents approximately 1.5 years, while a full recovery was observed after approximately a year for paths C and D. Intra-colonial variability in the response to heat stress with patchy bleaching, recovery and partial mortality has been previously noted^58^. This heterogeneity in bleaching response could be due to for example spatially variable pigment distribution or variations in coral morphology inducing subtle differences in boundary layer flow regimes and hence differing sensitivities to external environmental changes^59^.

Despite differences in the magnitude and time period for recovery, the initial response to the stress event was consistent between paths and characterized by an increase in Sr/Ca. The increase in Sr/Ca during stress events while likely linked to a decrease in the Ca^2+^-ATPase activity^20^, is only apparent for path B (Fig. 5), as the other two paths analysed for TE’s (C and D) showed minimal changes in [Ca^2+^] during the summer of 1998. The only consistent response between all sample paths was the increase in the proportion of Ca remaining in the cf after calcification is completed following the 1998 event, which might indicate a decrease in the precipitation efficiency. A decrease in precipitation efficiency could in turn help explain the reduction in extension rates (Fig. 2) as it might represent a slow-down of the calcification process; this highlights the importance of other processes besides pH up-regulation in coral calcification. While the seasonality in modelled *P* and [Ca^2+^]_cf_ are clearly explained by temperature changes (Supplementary Fig. S3) the seasonality present in residuals, Δ*P* and ΔCa (Fig. 5) suggest that light or other environmental parameter could also affect [Ca^2+^]_cf_. Seasonal changes to light could play a secondary role in controlling [Ca^2+^]_cf_ either through direct activation of the Ca^2+^ATPase enzyme or from energy (ATP) availability linked to the activity of the zooxanthella^60^.

### Boron isotopes as a proxy of thermal stress events

The coral δ^11^B (pH_cf_) showed two different responses to the 1998 thermal stress event (Figs 3 and 7): along path B values remained 0.1–1.1‰ (0.02 to 0.1 pH units) lower than typical values for an entire year, while paths A, C and D showed no significant change during the summer of 1998, but were characterized by a positive anomaly during autumn and winter of 1998. This intra-colonial δ^11^B variability indicates that the average response to thermal stress from bulk sampling could vary from a positive to a negative anomaly depending on the period sampled, and most likely, the degree of stress. This could help explain differences between previous studies^21, 31^, as corals may exhibit a variable δ^11^B response related to their degree of bleaching. For example, here for non-thermal stress years we observed a seasonality for δ^11^B of ~1‰ over a 6 °C range; therefore, the 0.8–1.5‰ decrease associated with an 8 °C increase reported by Dishon, *et al*.^31^ could be part of the normal response of the coral to seasonal temperature changes rather than a stress response. Having constraints on δ^11^B seasonal variations prior to the period of stress period is thus clearly essential to establish a proper baseline against which the magnitude of anomalies during stress events can be referenced.

In an earlier study^37^, a ~2.3‰ decrease in δ^11^B around 1998 that lasted for 4 to 5 years was observed in a coral from Arlington Reef, a mid-shelf reef in the GBR. The magnitude of this δ^11^B anomaly and lengthy recovery could reflect the different responses of individual coral colonies to bleaching or the result of different degrees to stress. For example, it is possible that the coral from Arlington^37^ bleached completely while the coral analyzed here was only partially bleached, which could have affected the observed recovery times and the severity of the δ^11^B anomaly recorded. Although it is not known whether the corals from either study bleached or not during 1998, the reduction in coral growth, scaring and anomalies in the geochemical signal observed here are clear evidence that the coral suffered severe stress of some nature. On the other hand, an examination of the Arlington core reveals that 1998 was not particularly unusual in terms of extension rates and no anomaly is evident in the Mg/Ca data (Supplementary Fig. S7). Nevertheless, this is consistent with observations that during 1998 inshore reefs of the GBR had higher levels of bleaching compared to outer reefs^61^. The variety of results between studies indicates a complex response of corals to thermal stress complicating the use of δ^11^B as a proxy for thermal stress.

### Intra-colonial and seasonal and variability in the pH and carbonate parameters of the calcifying fluid

The differences in the baseline pH_cf_ (δ^11^B) values of samples from different growth axis (Figs 3, 6 and 7) translated to pH values ~0.1 units lower along the “main” growth axis (paths B and D) compared to samples from a secondary or less ideal growth axis (paths A and C). It is not entirely surprising to find variations between sample paths as the composition for some trace elements have also shown to vary along different growth axis^14^ and along paths with suboptimal or disorganized skeletal architecture^62^. Differences in pH_cf_ were identified along different growth axis for *Stylophora pistillata* corals^28^; but with the apparent opposite sign, e.g. lower pH_cf_ in areas of lateral growth (sub-optimal) compared to areas of apical growth (optimal). However, our results are consistent with reports of higher δ^11^B values along off-axis compared to values from the primary growth axis for *Porites cylindrica* ^63^. These results suggest that coral physiology plays an important role in the coral δ^11^B and pH_cf_, which is likely linked to variations in growth in broad agreement with the IpHRAC model^10^. The variation between sample paths has important implications for paleo-reconstructions because in order to generate robust δ^11^B reconstructions an overlap of several samples should be obtained to confirm that no bias is introduced from moving from one sample axis to another one.

In terms of the ~1‰ seasonality found in coral δ^11^B (equivalent to ~0.1 pH units; Fig. 6) this is similar to that observed in *Porites* corals from Fanning Island in the equatorial Pacific^64^, Flinders Reef in the Coral Sea^27^, the GBR^12^ and Coral Bay in Western Australia^12^. The Ω_cf_ inversely related to changes in pH_cf_, with higher values in summer and lower values in winter, a priori appears to be consistent with reef-wide seasonal variations in seawater Ω and pH^65^. But while the seasonal changes in pH_cf_, DIC_cf_ and Ω_cf_ were in phase with the modelled changes in pH_SW_ and Ω_SW_ (Fig. 7), the seasonal amplitude in the coral was ~×2 and ~×30 larger, for pH_cf_ and DIC_cf_ respectively, than those changes in seawater. Furthermore, as experimental results indicate that corals mitigate the external changes in seawater pH^11, 17, 28, 63^ the large seasonal variability in coral δ^11^B observed in massive *Porites* was unexpected. The large seasonal variability in pH_cf_, DIC_cf_ and Ω_cf_ is therefore reflecting a strong physiological control of the carbonate parameters in the calcifying fluid^12^.

Some of the factors that could affect the pH_cf_ and DIC_cf_, and therefore Ω_cf_, include the transport of bicarbonate ion, diffusion of CO_2_, transport of seawater, proton pumping (Ca^2+^ATPase) and changing calcification rates^9, 32, 66, 67^. Higher photosynthesis^68^ and respiration rates^69^ in summer would likely result in a larger pool of DIC available for calcification in the form of CO_2_ and HCO_3_^−^, compared to winter. This means that the coral could potential convert more CO_2_ and thereby more HCO_3_^−^ to CO_3_^2−^. However, the amount of H^+^ the Ca^2+^ATPase can pump might be limited. For example once a threshold values in pH or Ω is passed the Ca^2+^ATPase enzyme appears to operate at a constant level^60^, with a similar behaviour observed for light level or photosynthesis rates^70^. If the diffusion of CO_2_ and transport of HCO_3_^−^ into the calcifying fluid increases in summer from higher respiration rates, the total of CO_2_ and HCO_3_^−^ converted to CO_3_^2−^ would be higher in summer than in winter. However, given a larger DIC pool in summer the Ca^2+^ATPase enzymes could reach substrate (H^+^) saturation. The latter scenario suggests that the proportion of CO_2_ and HCO_3_^−^ converted to CO_3_^2−^ in summer could be lower than that in winter due to larger summer pool of available DIC. This would results in higher summer DIC_cf_ but lower pH_cf_, explaining the observed seasonal variability in corals. Interestingly the higher Ω_cf_ observed in summer points to the generally more dominant role of DIC_cf_ enrichment in determining Ω_cf_.

Alternatively, given a greater DIC pool available for calcification in summer the coral could be actively reducing the pH_cf_, by for example decreasing the activity of the Ca^2+^ATPase enzymes, and hence limit the rate of conversion of HCO_3_^−^ into CO_3_^2−^ and thereby ‘control’ the rate of calcification^12^. In contrast, in winter the pH is increased to promote calcification from a reduced DIC pool in an attempt to maintain near-constant year-round calcification rates^12^. This later scenario appears to be supported by the lower [Ca^2+^] in summer (compared to winter) estimated from the trace element data (Supplementary Fig. S3). Regardless of the controlling mechanism the pattern of lower summer pH values would be further reinforced by higher rates calcification rates and therefore enhanced production of CO_2_ during the summer, when temperatures, Ω_cf_ (Fig. 7) and organic matrix production are at their highest. The biological mediated mechanism proposed could still in part be responding to changes in seawater pH by reinforcing gradients that promotes the diffusion of CO_2_ and in some cases, such as confined lagoons, modifies the conditions of the seawater used for calcification. In this sense experimental studies have demonstrated that under stable conditions coral pH_cf_ does responds to external changes in seawater pH^11, 28^, but with limited sensitivity.

### Response of the calcifying fluid’s carbonate chemistry to thermal stress

While coral calcification rates showed marked decreases during and following the high temperature stress event of the summer of 1998, the boron isotope and B/Ca data indicate that, despite some differences between sample paths, overall the coral continued to up-regulate pH and DIC (Fig. 7). These results indicate that a minimum threshold Ω value, through the up-regulation of pH and DIC at cf, must be attained for calcification to proceed. In this sense coral skeletons are therefore incomplete recorders of the complete thermal history that caused the stress event, only recording conditions when calcification actually occurs such as during the recovery phase. This was true even for path B, sampled adjacent to an area with signs of partial mortality where pH_cf_ remained elevated in comparison to seawater (Fig. 7). This not only supports the notion that coral pH up-regulation is energetically inexpensive^10^, but also highlights the importance of up regulation for calcification.

The elevation of pH and DIC and therefore Ω at the site of calcification is considered a critical step to promote coral growth/calcification. Although variations to the [Ca^2+^] in the cf linked to the activity of the Ca^2+^ATPase are unlikely to have a significant effect on coral growth since [Ca^2+^] is in excess^71^, the removal of ×2H^+^ and addition of [Ca^2+^] by Ca^2+^ATPase plays a crucial role in the elevation of the pH_cf_ and therefore Ω_cf_^8^. A reduction in the activity of Ca^2+^ATPase, as suggested to occur during thermal stress events^20^, is expected to lower pH and Ω_cf_; however, this is inconsistent with our findings of high pH_cf_, DIC_cf_ and hence Ω_cf_ following the 1998 event (Fig. 7). The high pH_cf_ and normal Ca^2+^ values (Fig. 5) for path C and D following the 1998 event suggest that, except perhaps for path B (near mortal conditions), the operation of the Ca^2+^ATPase pump coral was not subject to energy limitations. Therefore, the presence of apparently favourable pH_cf_ and Ω_cf_ conditions for calcification suggest that although pH up-regulation is an essential pre-requisite, other factors were responsible for the severe decrease in coral growth (calcification) evident along all paths following the 1998 stress event.

Calcification is a strongly controlled, spatially heterogeneous process that appears to take place in small pockets (~5 μm in length)^72, 73^. During thermal stress the number of active calcification spaces (pockets) could have been reduced due to lower supplies of metabolic sourced carbon, since in *Porites* corals this primarily depends on photosynthetically fixed carbon^30^. As such the recovery of bleached *Porites* corals (both massive and branching) depends on finite energy reserves^29^. Given that the primarily source of DIC is compromised, bleached *Porites* corals also appear to reduce respiration rates during the recovery phase in an attempt to maintain energy reserves^30^. Lower metabolic sourced DIC could lead to an increase in the time it takes to reach threshold levels of Ω_cf_ for calcification or a reduction in the number of active spaces of calcification at a given time, so that the up-regulation of pH_cf_ and DIC_cf_ represent the pre-requisite minimum Ω_cf_ conditions necessary for calcification. In turn, a reduction of the active spaces of calcification or time to up-regulate (effectively the active area of calcification) will reduce the rate of skeletal growth. Furthermore, a reduction in the organic matrix synthesis, a process also possibly linked to symbiont activity^74^ could affect crystal nucleation and growth rates^75^. Lower crystal nucleation and growth could translate into a reduction of calcium precipitated from a batch of calcifying fluid. A change in the synthesis of organic matrix, numbers of active sites of calcification or time required to up-regulate pH_cf_ and DIC_cf_ to reach threshold levels of Ω_cf_ are not mutually exclusive. In fact the variable response to stress recorded along different sample paths points towards multiple factors controlling changes in the composition of calcifying fluid.

In summary multi-proxy geochemical analysis of the coral skeleton including boron isotopes and trace elements is a powerful tool that can be used to more fully reconstruct the changes at the calcifying fluid. The response of the calcifying fluid in a *Porites* colony affected by thermal stress differed along sampled paths but ultimately translated into a reduction in growth of 1 to 2 years. This consistent decrease in linear extension rates is important as changes in growth and calcification could affect the coral’s ability to compete for space^2^ or increase the susceptibility to physical damage^76^. We also observed that coral up-regulation of pH_cf_ and DIC_cf_ and hence Ω_cf_ continued even under severe stress, suggesting that: (1) up-regulation of pH_cf_ is not energetically costly, (2) that there is a minimum Ω_cf_ for calcification to occur, and (3) that the conditions at the site of calcification are not the only factors responsible for the changes in coral growth. Changes in the effective active calcifying volume due to a reduction in respired DIC, and limits in the synthesis of organic matrix, both linked to the reduced activity of zooxanthellae, are likely to be critical factors that lead reduction in rates of coral calcification under thermal stress. These findings have important implications for the future of corals because despite their ability to up-regulate key carbonate parameters with increasingly frequent periods of thermal stress, severely compromising the ability of corals to maintain sustainable growth rates.

## Electronic supplementary material

Supplementary Online Material for: Response of coral calcification and calcifying fluid composition to thermally induced bleaching stress
