# Supplementary material for: Response of coral calcification and calcifying fluid composition to thermally induced bleaching stress
Source: Sci Rep. 2017 May 19;7:2207. doi: 10.1038/s41598-017-02306-x (PMC5438395; doi:10.1038/s41598-017-02306-x)
Supplement: Supplementary file 1 — Supplementary Online Material for: Response of coral calcification and calcifying fluid composition to thermally induced bleaching stress [file 41598_2017_2306_MOESM1_ESM.pdf]

Supplementary Online Material for:

Response of coral calcification and calcifying fluid composition to thermally induced bleaching stress

D'Olivo, J.P.[1]*, McCulloch, M.T.[1]

[1]ARC Centre of Excellence for Coral Reefs Studies, Oceans Institute and School of Earth Sciences, The University of Western Australia, Crawley 6009, Australia

* corresponding author: juan.dolivocordero@uwa.edu.au

This file includes:

- Text
- Table S1
- Figs. S1 to S7
- References

Estimates of seawater pH, DIC and Ω

The bimonthly seawater pH, DIC and Ω (pH_{SW}, DIC_{SW} and Ω_{SW}) records presented in Figure 7 were modeled based on instrumental SST, an average pCO_2 of 431 ppm with a seasonal variation of 60 ppm, and a seawater end member with summer total alkalinity (TA) of 2270 mmol kg SW^{-1} and winter TA of 2256 mmol kg SW^{-1}[1]. Calcium concentration in the cf was calculated assuming that the Ca^{2+}ATPase transported Ca but was specific against Sr. Seawater dissolved inorganic carbon (DIC_{SW}) changes were estimated from TA and pCO_2. For flood conditions the effects of dilution with a river water end member with TA of 787.7mmol kg SW^{-1} and DIC of 811.1 mmol kg SW^{-1} (DERM, 2014) were considered. Salinity and dilution factors were estimated based on the linear relationship described by D'Olivo, et al. [2] between the magnitude of past flood events and corresponding salinity values reported by King, et al. [3] and Walker [4]. Calculations were made using CO2SYS with carbonate constants K1 and K2 from Mehrback, et al. [5] refitted by Dickson and Millero [6], and for sulfate from Dickson [7] with 0 dbar pressure.

Estimates of Ca and *P* in the calcifying fluid

To calculate Ca_{cf} and P equation 2 (see main text) was expressed in terms of Sr/Ca and Mg/Ca where TE_{CF} was assumed to equal TE_{SW} (Mg_{SW} = 52.7mmol/Kg and Sr_{SW} = 0.087 mmol/Kg), the Kd_{TE} for Sr $Kd_{Sr} = e^{(-1.86+600/T_K)}$ [8] and for Mg Kd_{Mg}

$= e^{(-13+1700/T_K)}$, where T_K is the temperature in Kelvin. The resulting equations for Mg/Ca and Sr/Ca are therefore:

$$\left(\frac{\text{Mg}}{\text{Ca}}\right)_{arag} = \left(\frac{52.7}{\text{Ca}_{cf}}\right)\frac{\left(1 - P^{(-13+1700/\text{T}_K)}\right)}{(1-P)}$$

and

$$\left(\frac{\text{Sr}}{\text{Ca}}\right)_{arag} = \left(\frac{0.087}{\text{Ca}_{cf}}\right)\frac{\left(1 - P^{(-1.86+600/\text{T}_K)}\right)}{(1-P)}$$

This system of two equations (one for Sr/Ca and one for Mg/Ca) and two unknowns (Ca_{cf} and *P*) was solved simultaneously using the Matlab function fsolve for each pair of coral Sr/Ca and Mg/Ca data and corresponding *in situ* SST data.

The resulting modelled Ca_{cf} and *P* records showed a strong linear relationship with temperature (Figure S3 and Table S1). The parameters from the linear regression obtained between the modelled Ca_{cf} and *P* and SST from each sample path were used to estimate the proportion of Ca_{cf} and *P* explained by changes in temperature (Ca_{cf}–SST and *P*-SST) (Figure S4). These records were then used to estimate the Ca_{cf} and *P* anomalies presented in Figure 5 (ΔCa and Δ*P*) by subtracting the corresponding Ca_{cf}–SST or *P*-SST data from the Ca_{cf} or *P* data.

Table S1. Coefficients *m* and *b* for the linear equations of the form y = *m*x + *b* describing the relationship between temperature and the modeled $[Ca]_{cf}$ and *P* used to estimate the anomalies in Figure 5. Statistical parameters coefficient of determination and sample size included (all relationships statistically significant p<0.0001). Data from the 1998-1999 was not included in the calculations.

	$[Ca]_{cf}$			*P*		
	Path B	Path C	Path D	Path B	Path C	Path D
m	-0.064	-0.057	-0.056	-0.044	-0.044	-0.051
b	12.311	12.144	12.138	1.652	1.645	1.867
r^2	0.916	0.967	0.904	0.868	0.928	0.927
n	24	16	54	24	16	54

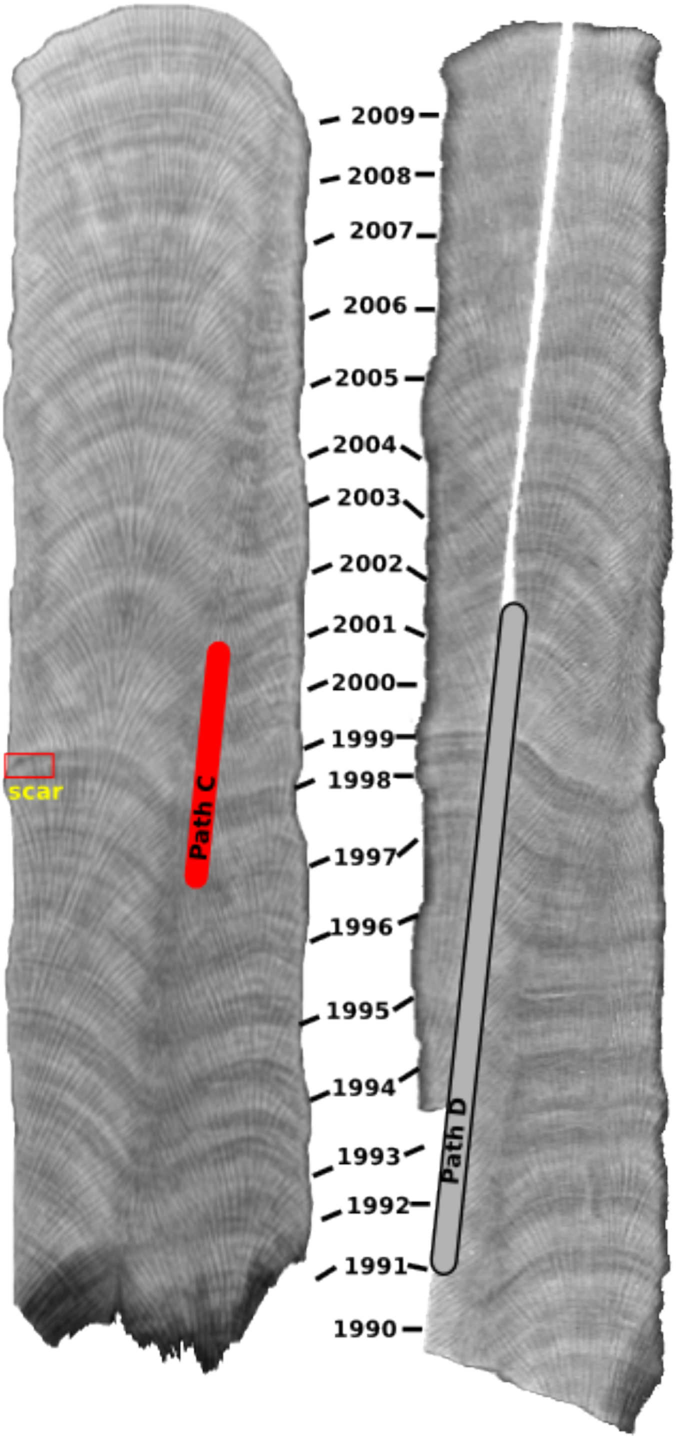

Figure S1. X-ray images of two slabs from core HAV09_3 displaying annual density bands. Sample paths for geochemical analysis are indicated.

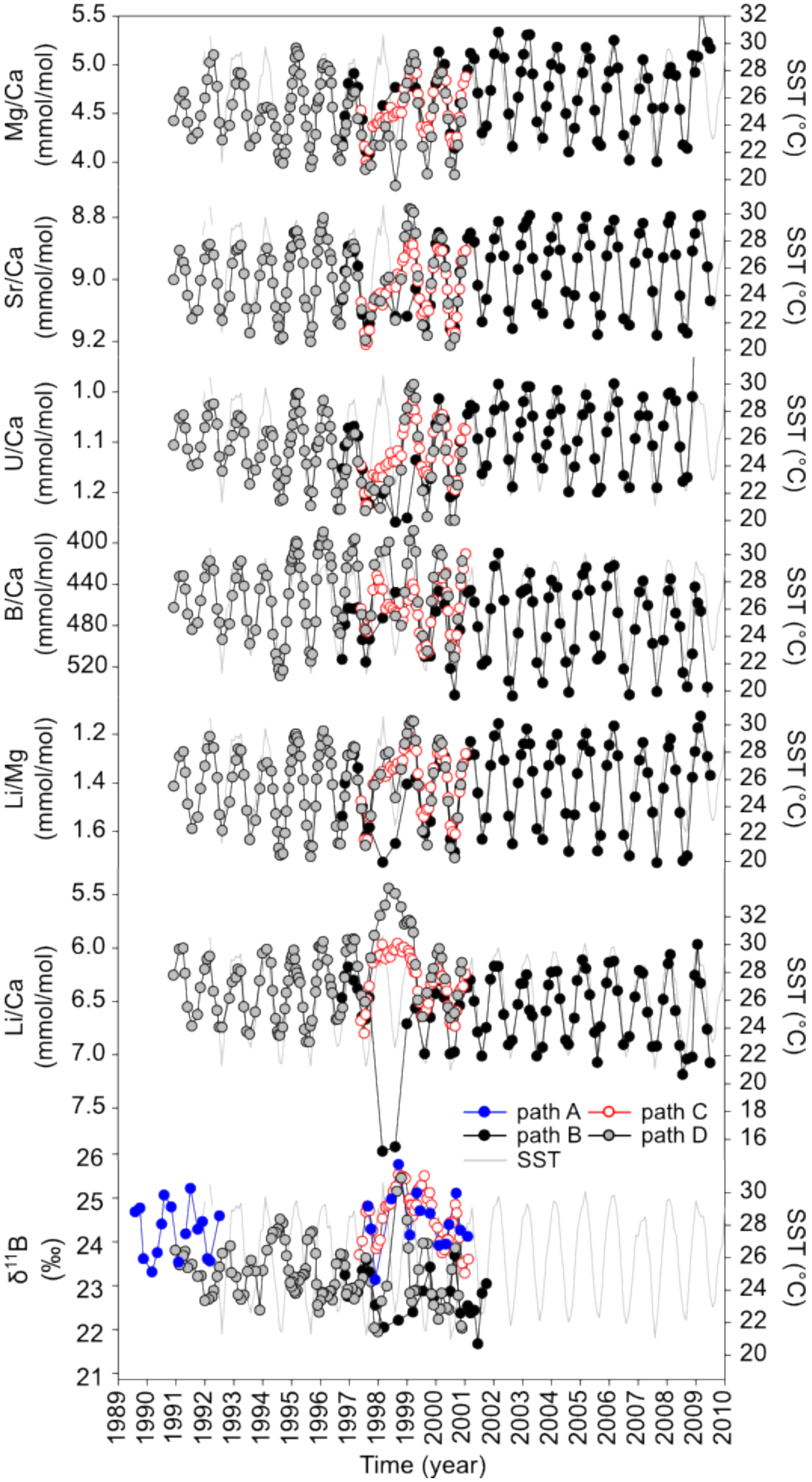

Figure S2. Untreated (uneven time distance) trace element and boron isotopes records for sample paths A (blue), B (black), C (red) and D (grey) compared to monthly SST.

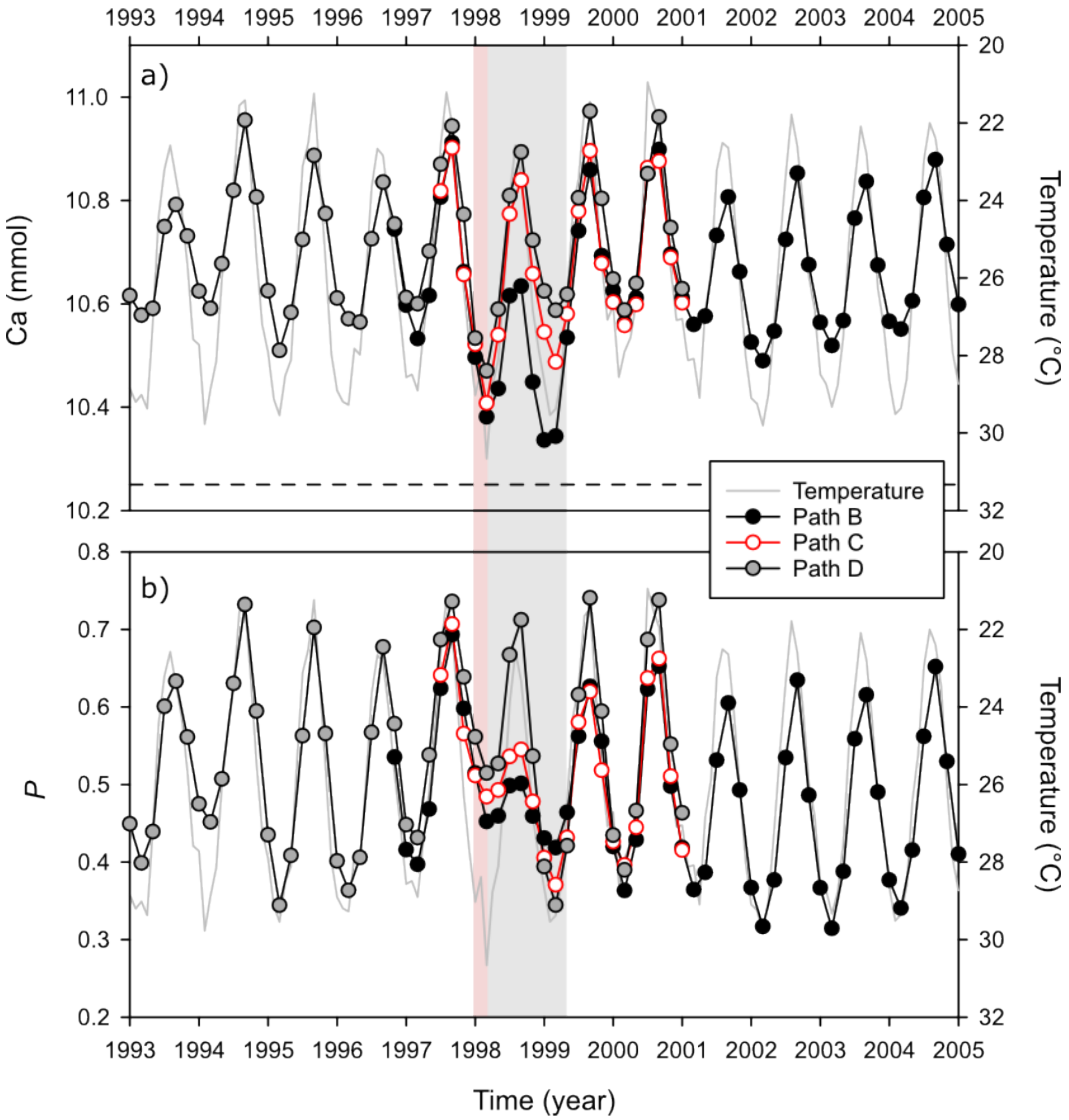

Figure S3. Comparison of *in situ* SST against modelled changes in: (a) the concentration of Ca^{2+} in the calcifying fluid and (b) the proportion of Ca^{2+} remaining in a batch of calcifying fluid after calcification finalizes (*P*). Data was calculated assuming the Ca^{2+}ATPase actively transported Ca into the calcifying fluid, but was selective against Sr and Mg. The horizontal dotted line indicates typical seawater values. The pink shaded area highlights the timing of the 1998 bleaching event, the grey shaded area highlights the recovery period. The temperature axis has been reversed to facilitate comparisons.

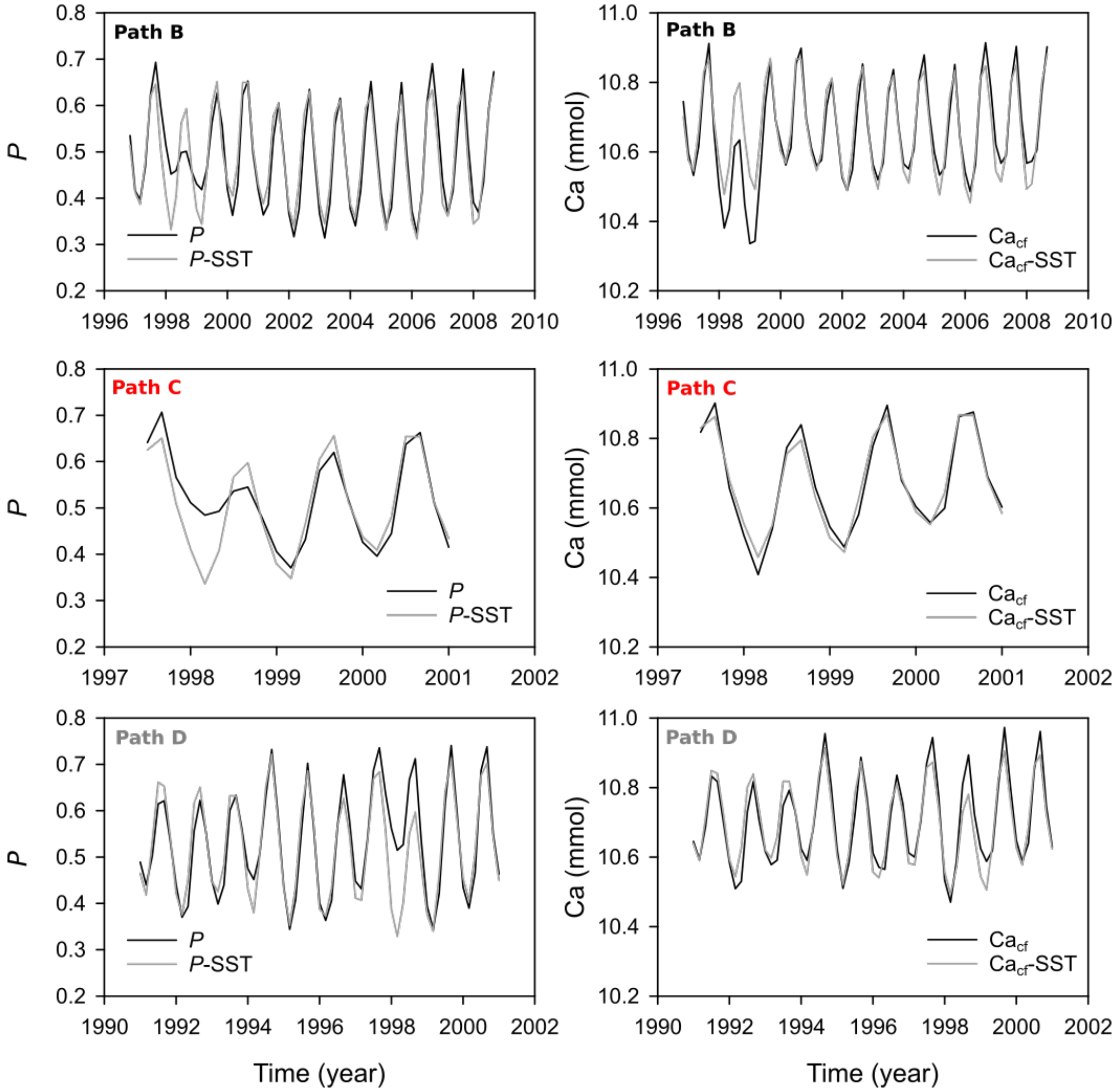

Figure S4. Comparison between modelled changes in $[Ca^{2+}]_{cf}$ and P (Figure S3) and against SST component of $[Ca^{2+}]_{cf}$ and P estimated from the relationships in table S1.

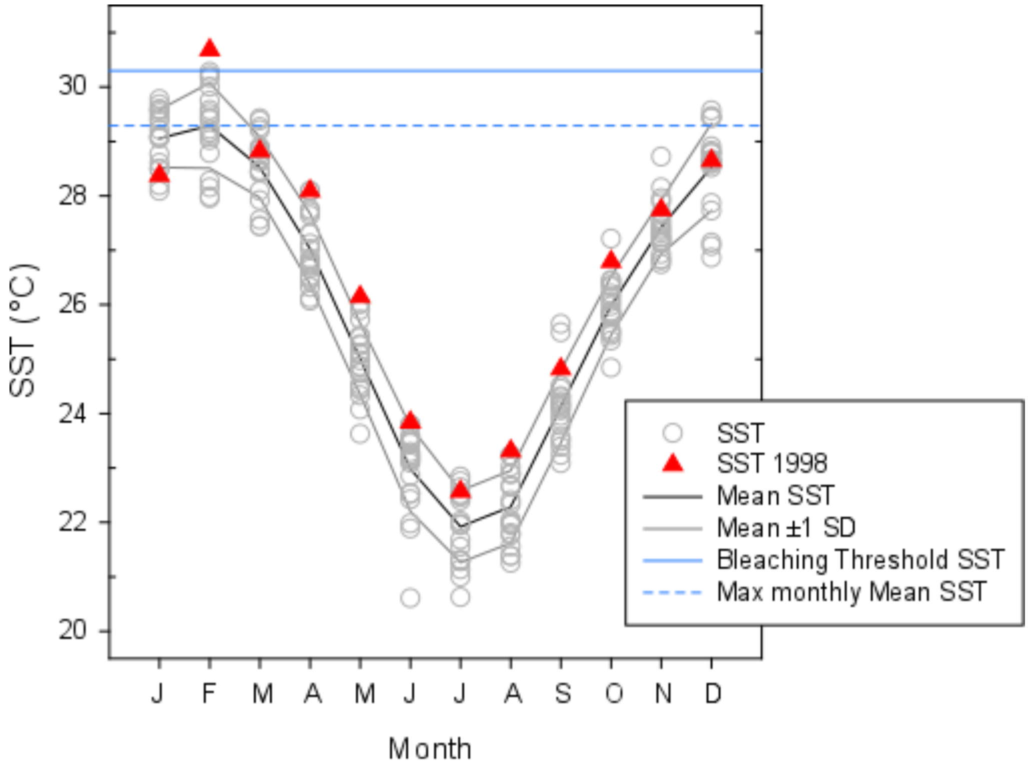

Figure S5. Monthly climatology and monthly mean climatology (average monthly values) for the *in situ* SST records. The horizontal discontinuous blue line indicates the maximum monthly mean *in situ* SST, the horizontal continuous blue line is the bleaching threshold (+1°C maximum monthly mean SST). The maximum monthly mean SST represents the warmest monthly mean value (CRW).

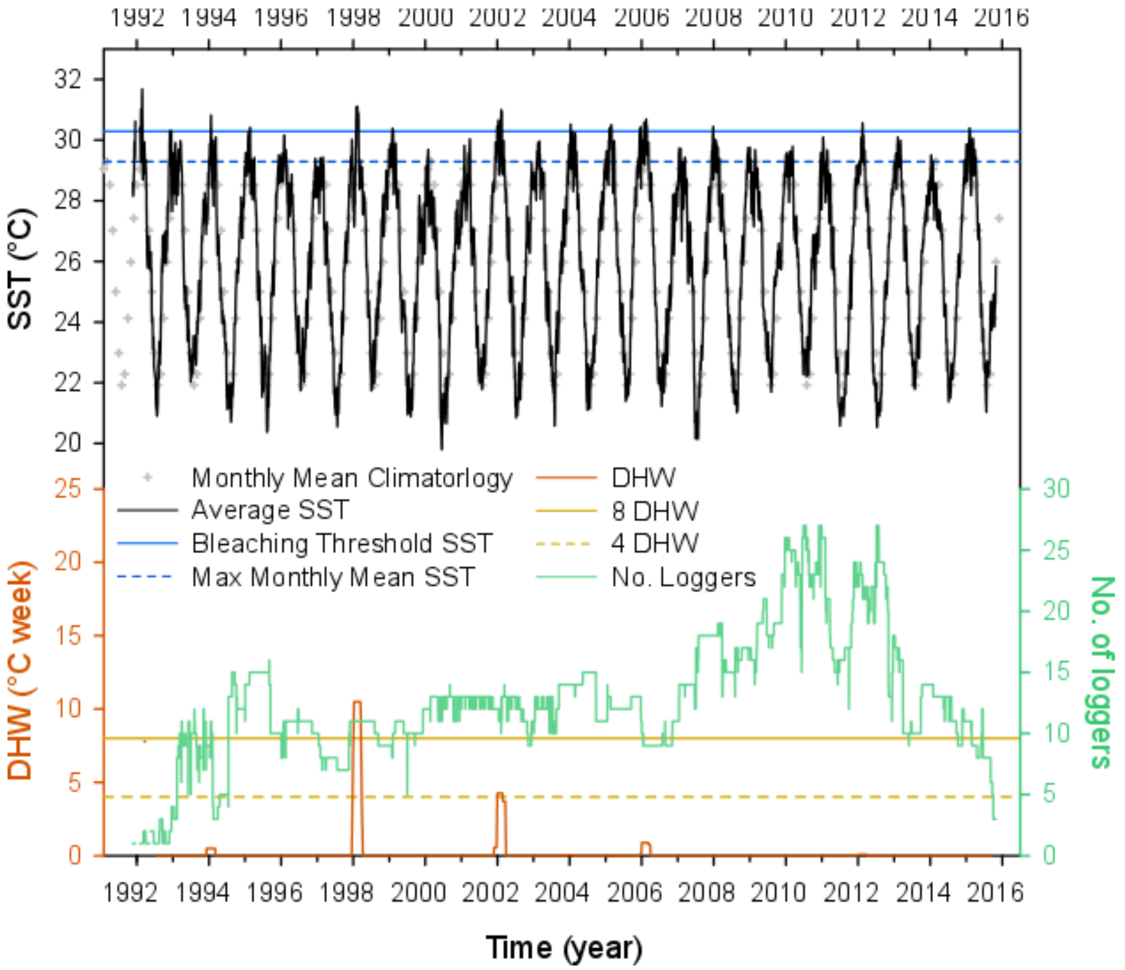

Figure S6. **Top panel** - Records of daily *in situ* SST and monthly mean climatology (average monthly values) the horizontal discontinuous blue line represents the maximum monthly mean *in situ* SST, the horizontal continuous blue line is the bleaching threshold (+1°C maximum monthly mean SST). **Bottom panel** - Estimate of the accumulation of thermal stress calculated as degree heating weeks (DHW). The yellow discontinuous line indicates a bleaching alert level 1 (4 <= DHW < 8), the continuous yellow line indicates a bleaching alert level 2 (8 <= DHW). Also shown are the number of temperature loggers.

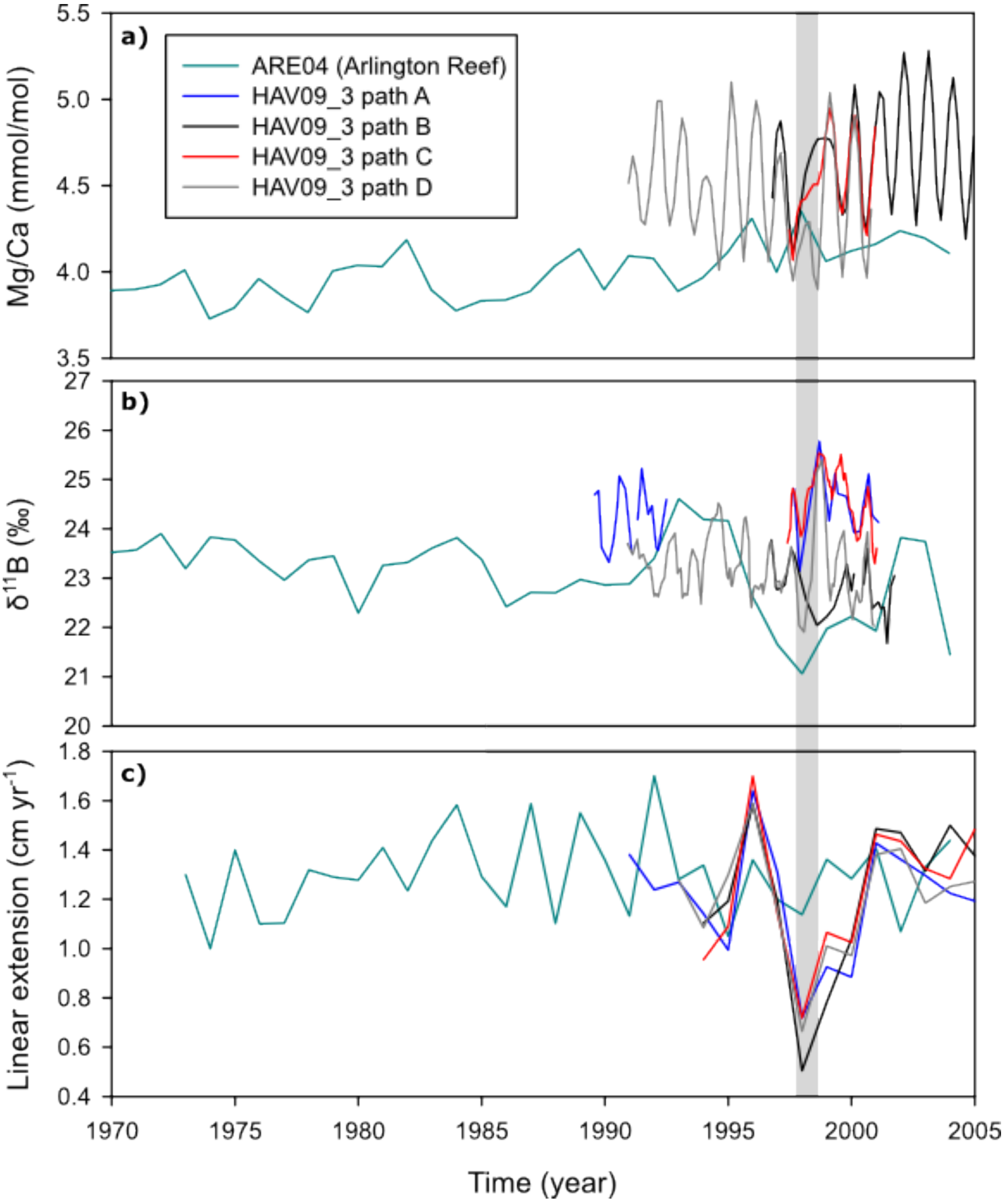

Figure S7. Comparison between the Mg/Ca (a) boron isotopes (b) and annual linear extension (c) for *Porites* coral records from Arlington Reef (ARE04)[9] and from Havannah Island (HAV09_3; this study).

References

1 Uthicke, S., Furnas, M. & Lonborg, C. Coral reefs on the edge? Carbon chemistry on inshore reefs of the great barrier reef. *PloS one* **9**, e109092, doi:10.1371/journal.pone.0109092 (2014).

2 D'Olivo, J. P., McCulloch, M. T., Eggins, S. M. & Trotter, J. Coral records of reef-water pH across the central Great Barrier Reef, Australia: assessing the influence of river runoff on inshore reefs. *Biogeosciences* **12**, 1223-1236, doi:10.5194/bg-12-1223-2015 (2015).

3 King, B., McAllister, F., Wolanski, E., Done, T. & Spagnol, S. River plume dynamics in the central Great Barrier Reef in *Oceanographic Processes of Coral reefs: Physical and Biological Links in the Great Barrier Reef* (ed E. Wolanski) 145–160 (CRC Press, 2001).
4 Walker, T. Seasonal Salinity Variations in Cleveland Bay, Northern Queensland. *Aust. J. Mar. Fresh. Res.* **32**, 143-149 (1981).
5 Mehrback, C., Culberson, C. H., Hawley, J. E. & Pytkowicz, R. M. Measurement of the apparent dissociative constants of carbonic acid in seawater and atmospheric pressure. *Limnol. Oceanogr.* **18**, 897-907 (1973).
6 Dickson, A. G. & Millero, F. J. A Comparison of the Equilibrium-Constants for the Dissociation of Carbonic-Acid in Seawater Media. *Deep-Sea Res.* **34**, 1733-1743 (1987).
7 Dickson, A. G. Thermodynamics of the dissociation of boric acid in synthetic seawater from 273.15 to 318.15 K. *Deep-Sea Res.* **37**, 755-766 (1990).
8 Sinclair, D. J. RBME coral temperature reconstruction: An evaluation, modifications, and recommendations. *Geochim. Cosmochim. Ac.* **154**, 66-80, doi:10.1016/j.gca.2015.01.006 (2015).
9 Wei, G., McCulloch, M. T., Mortimer, G., Deng, W. & Xie, L. Evidence for ocean acidification in the Great Barrier Reef of Australia. *Geochim. Cosmochim. Ac.* **73**, 2332-2346, doi:10.1016/j.gca.2009.02.009 (2009).
